# Experimental Investigation on Form Error for Slow Tool Servo Diamond Turning of Micro Lens Arrays on the Roller Mold

**DOI:** 10.3390/ma11101816

**Published:** 2018-09-25

**Authors:** Yutao Liu, Zheng Qiao, Da Qu, Yangong Wu, Jiadai Xue, Duo Li, Bo Wang

**Affiliations:** Centre for Precision Engineering, Harbin Institute of Technology, 92 West Dazhi Street, Nan Gang District, Harbin 150001, China; 16B908095@stu.hit.edu.cn (Y.L.); qiaozhengyunlong@126.com (Z.Q.); bennyqu007@yahoo.com (D.Q.); yuguanzi@163.com (Y.W.); brucexjd@hit.edu.cn (J.X.)

**Keywords:** slow tool servo, ultra-precision diamond turning, micro lens arrays (MLAs), chatter mark, forming method

## Abstract

Slow tool servo (STS) assisted ultra-precision diamond turning is considered as a promising machining process with high accuracy and low cost to generate the large-area micro lens arrays (MLAs) on the roller mold. However, the chatter mark is obvious at the cut-in part of every machined micro lens along the cutting direction, which is a common problem for the generation of MLAs using STS. In this study, a novel forming approach based on STS is presented to fabricate MLAs on the aluminum alloy (6061) roller mold, which is a high-efficiency machining approach in comparison to a traditional method based on STS. Based on the different distribution patterns of the discrete point of micro lens, the equal-arc method and the equal-angle method are also proposed to generate the tool path. According to a kinematic analysis of the cutting axis, the chatter mark results from the overlarge instantaneous acceleration oscillations of the cutting axis during STS diamond turning process of MLAs. Cutting parameters including the number of discrete points and cutting time of every discrete point have been experimentally investigated to reduce the chatter mark. Finally, typical MLAs (20.52-μm height and 700-μm aperture) is successfully machined with the optimal cutting parameters. The results are acquired with a fine surface quality, i.e., form error of micro lenses is 0.632 μm, which validate the feasibility of the new machining method.

## 1. Introduction

In recent years, microstructure surfaces, such as micro lens arrays (MLAs) with spherical shape and accurate position, have been extensively used in a series of areas, including liquid crystal display (LCD), illumination, data storage, sensor devices, and so forth, due to their excellent optical features [1,2,3,4]. Therefore, many researchers have paid attention to exploring more controllable and efficient methods to fabricate MLAs. Investigations showed that the roll-to-roll (RTR) replication technique [5,6,7,8], which is a typical continuous forming process, is regarded as a promising approach with high accuracy, high efficiency, and low cost, to generate various microstructure surfaces in comparison with plane replication technology. The cylindrical roller mold containing millions of micro lenses plays a key role in the RTR technique. Although various fabrication methods have already been proposed, including chemical etching [9], laser lithography [10], diamond broaching [11], and end-fly-cutting [12], the methods mentioned are not suitable for fabricating MLAs on the cylindrical roller mold due to their limitations. Micro-milling is also used for fabricating various microstructures on flat or roll molds [13,14,15,16], which can cut nonferrous metal and tool steel. But machining efficiency of the micro-milling process is too low to fabricate large-area micro lens arrays.

Up to now, ultra-precision diamond turning using a slow/fast tool servo is regarded as a state-of-the-art method to generate MLAs on the cylindrical roller mold [17,18,19,20]. To fabricate MLAs with a high aspect ratio (AR), the clearance angle of the cutting tool has been increased via lowering the position of the cutting tool tip relative to the rotation center of the workpiece in the vertical direction [21]. Based on this novel machining method, the interference phenomenon between the flank surface of the diamond tool and the machined surface can be eliminated when MLAs with high AR are processed using slow tool servo (STS) diamond turning on the roller mold. As a consequence, the AR of MLAs can be increased to about five times larger than previous fabrication results with the method of traditional diamond turning using the same diamond tool. In addition, to solve the problem of cutting tool wear during large-area MLAs machining over a roller mold, Gao et al. [22,23] have developed a fabrication method of tool replacement stitching. The contact force between the cutting tool tip and the roll surface was utilized to scan the interrupted point on the roll surface by a force sensor integrated on the fast tool servo (FS-FTS) system. A sub-micrometer cutting tool positioning for the new replaced cutting tool can be realized derived from the proposed method. Kong et al. [24] have presented a novel orthogonal slow tool servo (OSTS) assisted diamond turning approach. In addition, a tool path generator based on OSTS has been developed for manufacturing different wavy microstructures. In such a manner, the various wavy patterns with fine surface finish can be machined stably on a roller surface. To fabricate the complex microwave pattern of optical films on a Ni-coated steel roller mold, Lee et al. [25] have developed a right/left-horizontal swing fast tool servo (HFTS) with different hinge structures. The simulated analysis and experimental results have shown that HFTS with a single fixed hinge is more appropriate for precision manufacturing of the optical microstructure than HFTS with double fixed hinges because the former can avoid over-constraint conditions. Both the OSTS and the HFTS machining approaches provide feasibility for the fabrication of various wave prism patterns.

Although sufficient research has been dedicated to the generation of microstructures using STS/FTS assisted diamond turning, most of these aimed to manufacture MLAs on the planar mold rather than the roller mold. And the trajectory method is always used for processing MLAs on the planar or cylindrical mold [18,26,27]. The productivity of STS diamond turning is restricted by the preceding tool path. What is more, the obvious chatter mark occurs at the cut-in part of every processed micro lens along the cutting direction, which is a general problem for machining MLAs using STS [21,28]. This defect reduces the surface quality of micro lens and affects its optical properties. But the problem has received relatively little attention in previous research.

In this study, a new machining method based on STS diamond turning is proposed to generate MLAs on the roller mold. Two distribution strategies of the discrete point are defined to generate the tool path. Furthermore, the effects of cutting parameters, such as the number of discrete points and cutting time of every discrete point on the chatter mark of micro lens surface, are analyzed in detail. Finally, a series of cutting experiments are carried out to validate the effectiveness of the proposed methodology and analysis.

## 2. A New Fabrication Approach of MLAs

### 2.1. Principle of Machining Method

**Figure 1:**
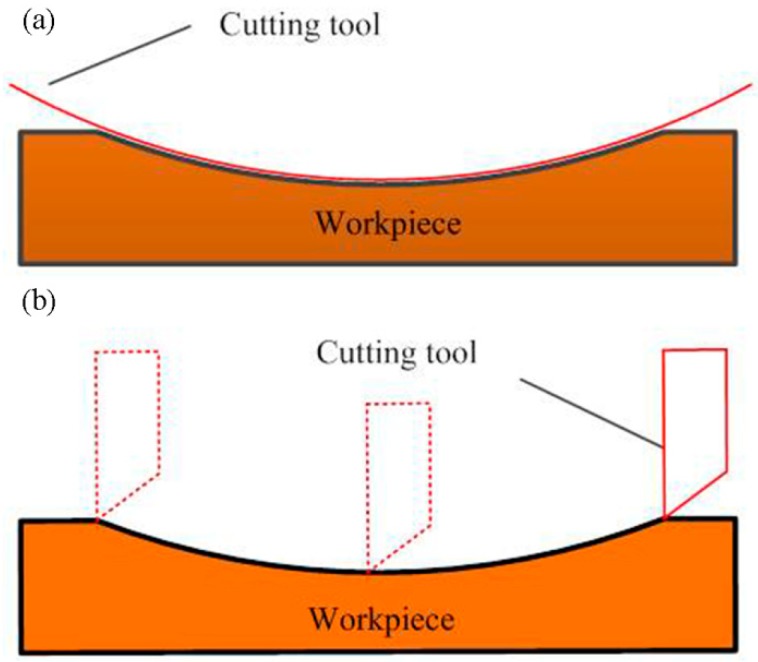
Schematic of the trajectory method. (**a**) The axial direction; (**b**) the circumferential direction.

**Figure 2:**
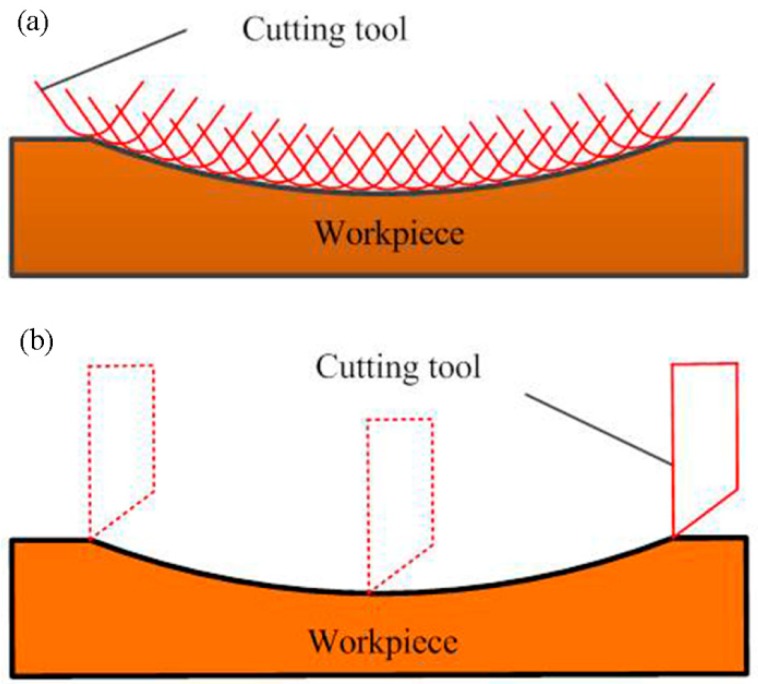
Schematic of the forming method. (**a**) The axial direction; (**b**) the circumferential direction.

In the conventional STS diamond turning process, trajectory method is usually adopted to fabricate MLAs on the planar or cylindrical mold. In other words, every micro lens is determined by multiple cutting to generate the complete profile at the axial and circumferential direction, as shown in Figure 1. Therefore, the application of the STS method is already restricted by the relatively low production efficiency of this machining method in the research field of optical manufacturing. To overcome the drawback associated with the traditional trajectory method, a novel and high-efficiency machining approach of MLAs on the roller mold is presented in this study. As illustrated in Figure 2, the forming method based on STS diamond turning was used for the generation of MLAs on the roller mold. Single cutting at the axial direction can process an entire micro lens, so it decreases the period of the machining process. The production efficiency of MLAs can be improved dozens of times in comparison with the conventional trajectory method based on STS diamond turning and micro-milling [14,27].

### 2.2. Tool Path Selection

**Figure 3:**
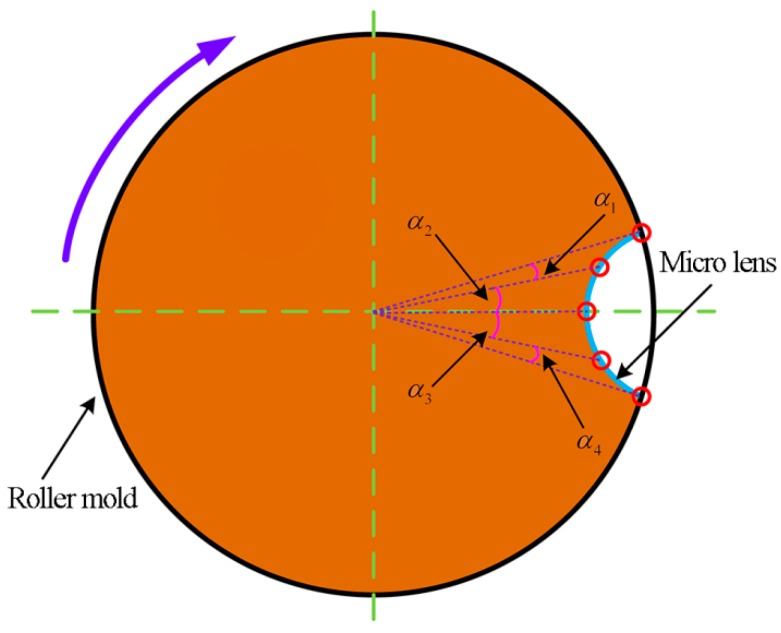
The equal-arc method.

To generate the tool path for the machining of MLAs on the roller mold, the equal-arc method and the equal-angle method are presented in this work, which is based on the various distribution patterns of the discrete point of micro lens. As for the equal-arc method, the arc length of micro lens between any two adjacent discrete points is defined to be equal. While the rotation angle of the C-axis from one discrete point to the next discrete point is different, i.e., $\alpha_{1}\neq \alpha_{2}$ and $\alpha_{3}\neq \alpha_{4}$, as shown in Figure 3. And cutting time of every discrete point is set as a constant value, which is determined by the machining parameters. Therefore, the velocity of the C-axis of the motion system is also varied between the different discrete points during processing. So, this motion control strategy will result in frequent acceleration and deceleration of the spindle during machining. Additionally, because the weight of the roller mold is normally hundreds of kilogram or even a ton, frequently changing speed would greatly affect the dynamic behavior and accuracy of the motion control in the case of the heavy load.

**Figure 4:**
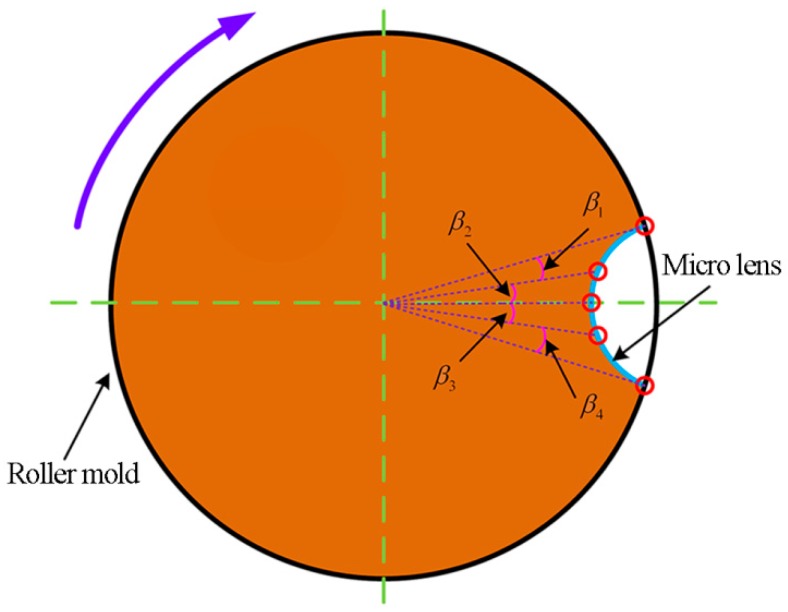
The equal-angle method.

To overcome the deficiency of the equal-arc method, the equal-angle method is developed in the subsequent study. In this motion control strategy, the rotation angle of the C-axis for adjacent discrete points is the identical value, i.e., $\beta_{1}=\beta_{2}$ and $\beta_{3}=\beta_{4}$, as shown in Figure 4. Therefore, the spindle has uniform motion between the different discrete points during processing. In terms of the control and motor system, such a strategy is considered more reasonable and easier to implement. So, the equal-angle method is utilized to fabricate MLAs in all experiments.

## 3. Experimental Setup

**Figure 5:**
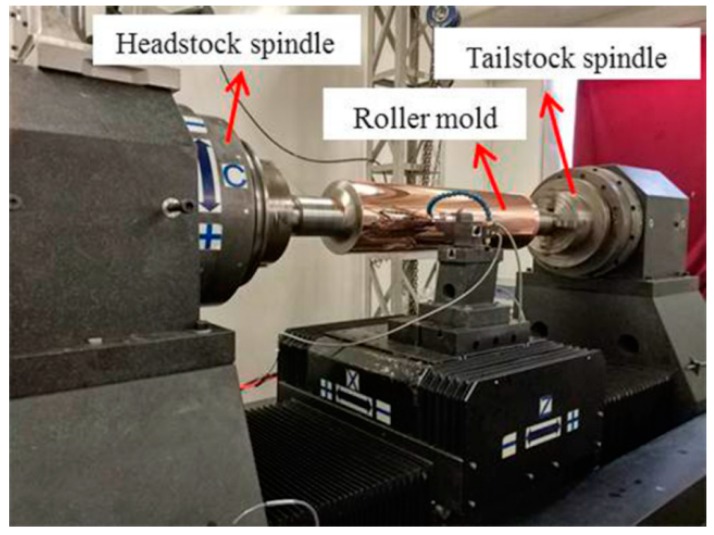
Experimental setup for the machining process.

**Figure 6:**
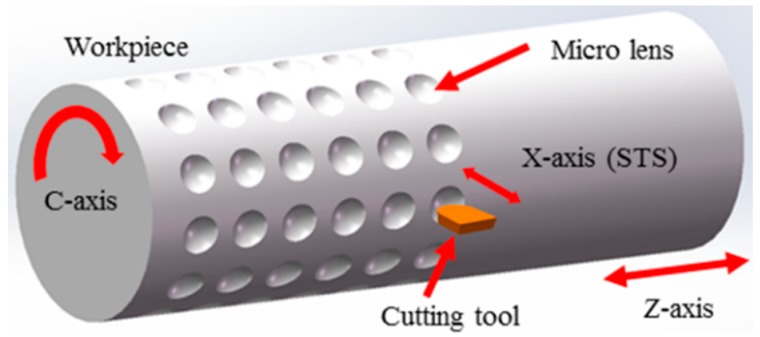
Schematic of slow tool servo (STS) diamond turning of micro lens arrays (MLAs).

To validate the effectiveness of the proposed new STS assisted ultra-precision diamond turning method, MLAs were generated using the in-house developed ultra-precision horizontal drum roll lathe (including the linear X-, Z-, and the rotatory C-axes) in this study, as shown in Figure 5. The specification of the ultra-precision drum roll lathe is summarized in Table 1. The machining principle of STS assisted ultra-precision diamond turning of MLAs is shown in Figure 6. Both ends of the roller mold are fixed on the headstock and tailstock spindle through four-jaw independent chuck, respectively. Among all those axes, the X-axis of the machine system is used as the cutting axis, which controls the cutting depth and aperture of a single micro lens. The pitch between two adjacent micro lenses in the radial direction is determined by the rotary motion of the C-axis. The pitch between two adjacent micro lenses in the axial direction, which is parallel to the centerline of the roller mold, is controlled by linear motion of the Z-axis. In addition, the X-axis and C-axis are synchronized to form the designed micro lens via the Universal Motion and Automation Controller (UMAC) in the circumferential direction. Therefore, the spindle speed is related to the diameter of roller mold, number of discrete points and cutting time of every discrete point. When a circle of micro lenses is finished on the roller mold, the Z-axis moves the distance of an axial pitch. Then the next circle of micro lenses can be processed sequentially.

Before the fabrication of MLAs, the surface of the roller mold must be machined to eliminate both imbalances from itself and installation error. Generally, this process consists of two parts, including rough machining and finish machining. In the first stage of rough machining, a cutting depth of 15 μm and a feed rate of 20 μm/rev are adopted to remove materials rapidly using a round-shaped polycrystalline diamond (PCD) tool. In the second stage for finish machining, a cutting depth of 5 μm and a feed rate of 5 μm/rev are applied to generate a mirror-like surface with a round-shaped natural diamond (ND) tool. Finally, MLAs are machined using the above-mentioned ND tool, which was used for the mirror finishing. The purpose of this operation is to avoid the error which results from repetitive tool setting. The machining accuracy could be affected by the changes of the cutting depth and the aperture of every micro lens, which are derived from the above tool setting error.

**Figure 7:**
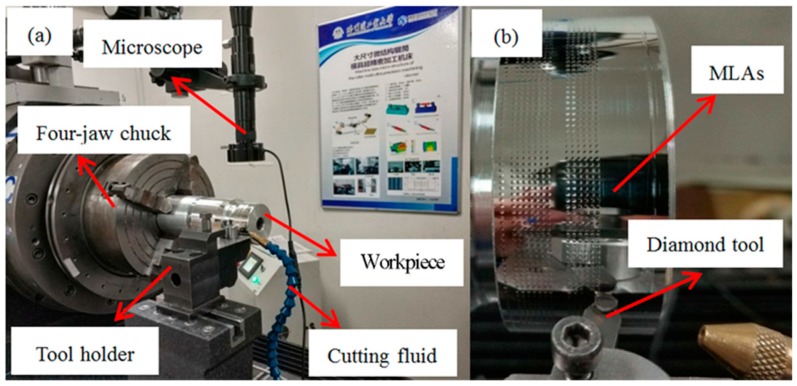
MLAs on the small workpiece. (**a**) The machining system; (**b**) partial enlarged view.

In terms of the MLAs experiments, the experimental conditions including the dimension of the cutting tool, cutting parameters and size of micro lens are listed in Table 2. In general, the size of the roller mold was too large to directly measure MLAs on its surface using existing measuring instrument in the laboratory. To solve this problem, MLAs with the same aperture were processed using the same cutting parameters by the above machine tool on a small roller mold, which could be directly inspected by commercial ultra-precision 3-D metrology devices, as shown in Figure 7.

## 4. Results and Discussion

### 4.1. Form Error of Micro Lens

**Figure 8:**
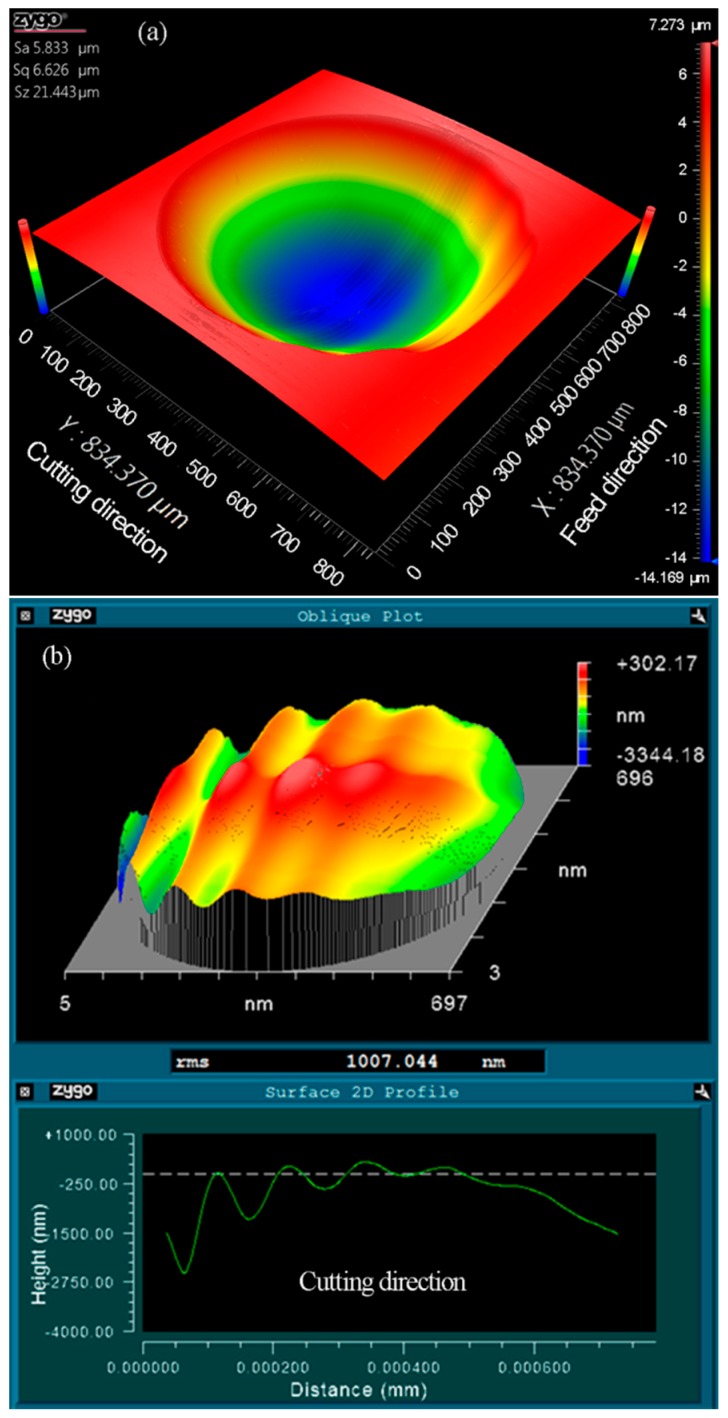
Zygo photographs of micro lens. (**a**) 3-D topography of machined micro lens; (**b**) The form error of machined micro lens surface from a designed sphere (*n* = 100, *t* = 1 ms).

As illustrated in Figure 8a, the three-dimensional topography of the machined micro lenses was measured using a white light interferometer (Zygo Newview 8200 from Zygo Corp., Middlefield, CT, USA). Wavy distortions can be clearly observed at the cut-in part along the cutting direction. The form error between the machined micro lens surface and an ideal spherical surface (*R* = 2.995 mm) was derived using a post-process software (Metropro, from Zygo Corp., Middlefield, CT, USA) and revealed in Figure 8b, appearing to obvious chatter mark in the same part. Owing to the location of both the distortion and the chatter mark which are a one-to-one correspondence, it is expected that the distortions of micro lens surface are caused by the chatter mark. It is a general problem with the fabrication of MLAs using trajectory method or forming method based on STS diamond turning.

**Figure 9:**
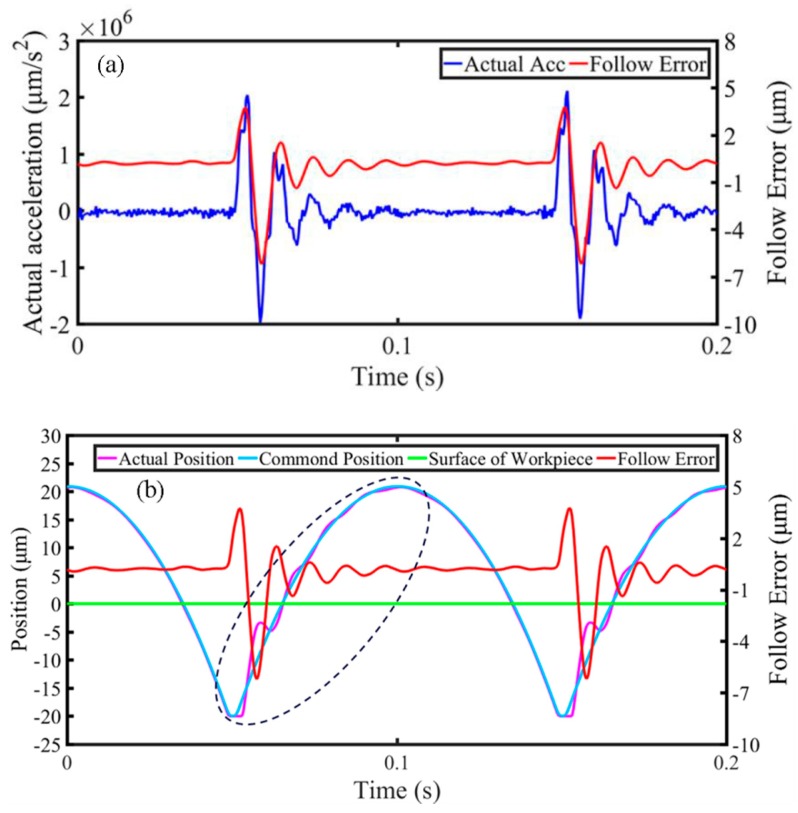
The collected real-time kinematic information of the cutting axis. (**a**) Actual acceleration and follow error; (**b**) Follow error, actual, and command position.

To investigate the generation of the chatter mark, the real-time motion information of the cutting axis (X-axis) was collected synchronously via the UMAC during processing, including actual acceleration, follow error, actual position, and command position, as revealed in Figure 9. In addition, the green lines as highlighted in Figure 9b represent the surface of the workpiece, i.e., the initial position of the cutting process for every micro lens. As observed in Figure 9a, there are a few overlarge acceleration oscillations occurring for the X-axis in the MLAs processing. Further, the fluctuations of the follow error are accordingly caused by the acceleration oscillations. It can be seen that the maximum amplitude of follow error is up to several micrometers, but the oscillation amplitude will decrease over time. Even so, once the large fluctuations of follow error occur in the cutting area, i.e., the upper part of the green line in Figure 9b, both the actual position and the command position of the cutting axis (X-axis) are obviously misaligned, as highlighted with a black dotted circle in Figure 9b. This phenomenon will result in the distortion of the profile for machined micro lenses. Thus, it can be determined that excessive follow error is reflected to the surface of micro lens in the form of a chatter mark. Further, the chatter mark can be attributed to an overlarge instantaneous acceleration and deceleration of the cutting axis (X-axis) of the machine tool during processing.

### 4.2. Effect of the Number of Discrete Points

**Figure 10:**
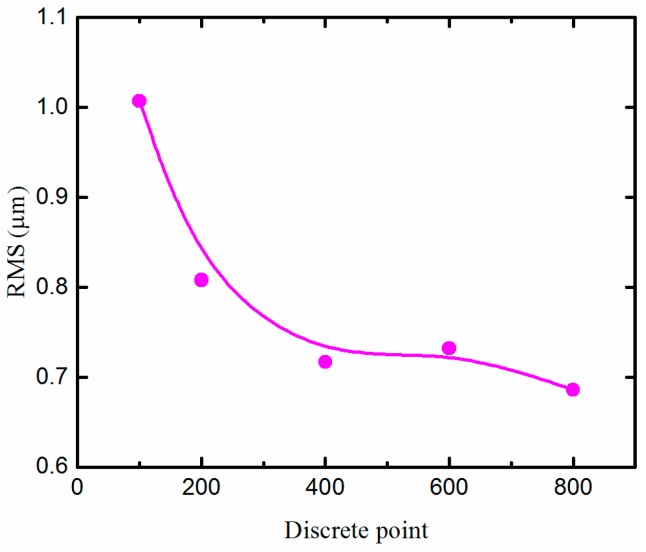
Form error of micro lenses machined at various discrete points.

In this section, the effects of the number of discrete points for the form error of micro lens will be investigated in detail. The corresponding experiments were carried out on the aluminum alloy (6061) roller mold using the STS approach based on the self-developed ultra-precision horizontal drum roll lathe. The number of discrete points is 100, 200, 400, 600, and 800 for a single micro lens, and the cutting time of every discrete point is 1 ms. The size of machined micro lenses is shown in Table 2. The plot of form error of machined micro lenses at a various number of discrete points is shown in Figure 10. In this study, Root-Mean-Square (RMS) is used for characterizing the form error of machined micro lenses. In addition, the measured data were processed using a Gaussian low-pass filter provided by Metropro before the calculation of RMS. The mathematical definition and the digital implementation of RMS can be formulated as:

(1) $$Z_{RMS}=\sqrt{\frac{1}{N}\sum_{n=1}^{N}{\left|z_{n}\right|}^{2}}$$

where *N* is the number of the measurement point of every micro lens and *z_n_* the form error of the profile at point number *n*.

**Figure 11:**
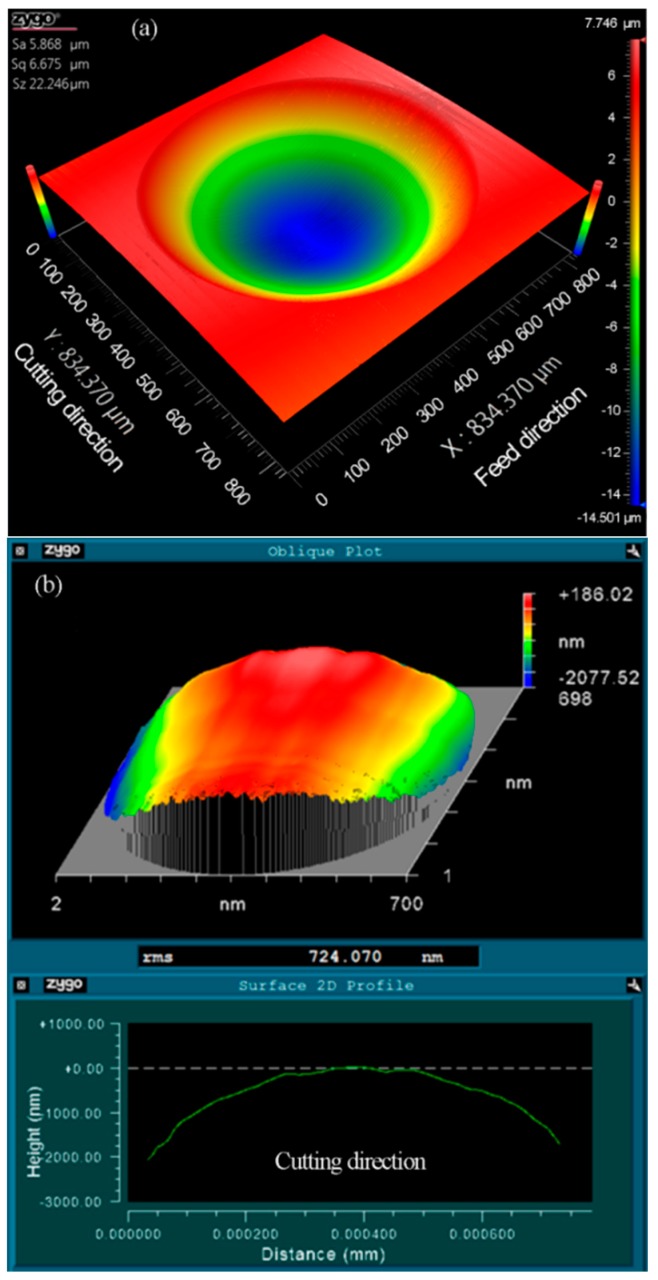
Zygo photographs of micro lens. (**a**) 3-D topography of machined micro lens; (**b**) The form error of machined micro lens surface from a designed sphere (*n* = 400, *t* = 1 ms).

As it can be seen from Figure 10, when the number of discrete points is 100, RMS is more than 1 μm. The shape of every micro lens distorts at the cut-in part due to the generation of the overlarge chatter mark, as shown in Figure 8b. With the increasing of the number of discrete points, RMS decreased obviously. Furthermore, MLAs have a fine surface quality with 0.724 μm RMS when the discrete point is 400, i.e., RMS is decreased by about 28.10%, as shown in Figure 11. No chatter mark appears at the cut-in part along the cutting direction. But the surface quality of MLAs could not be improved significantly when the discrete point is 800, i.e., RMS is reduced by about 31.88% (RMS = 0.686 μm).

According to the above experimental results, the surface quality can be improved effectively by increasing the number of discrete points of every micro lens. This is because as the number of discrete points increases, the cutting depth at each discrete point decreases accordingly. So, when the cutting time of every discrete point is a constant value, acceleration and deceleration of the cutting axis (X-axis) also reduce during processing. Then above overlarge acceleration oscillations of the cutting axis can be eliminated, and so do fluctuations of its follow error, when the number of discrete points is sufficient. Consequently, the micro lens with no surface chatter mark can be fabricated. It is noted that too many discrete points do not always reduce form error significantly. After the chatter mark disappears on the surface of micro lens, the number of discrete points is no longer the main factor affecting surface quality.

### 4.3. Effect of Cutting Time of Every Discrete Point

**Figure 12:**
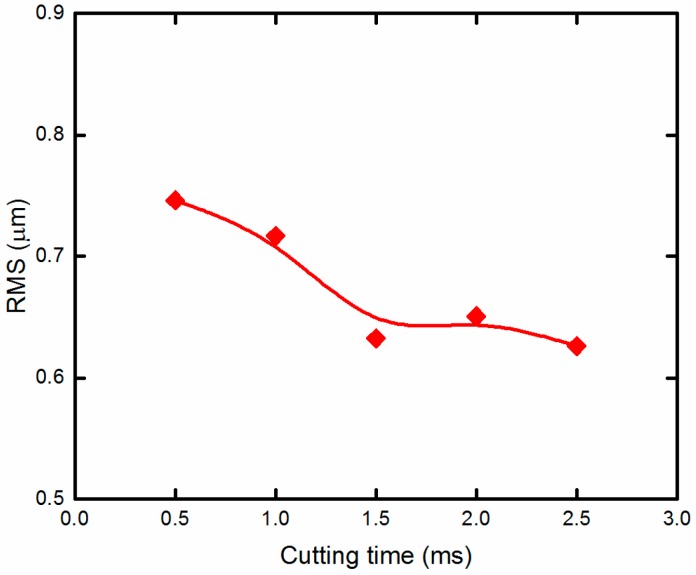
Form error of micro lenses machined at different cutting time of every discrete point.

The cutting time of every discrete point is another major cutting parameter for MLAs machining. Cutting time of every discrete point and the number of discrete points together determine the machining speed of the micro lens. To analyze the influences of the cutting time of every discrete point on form error of micro lens, some cutting experiments were conducted at various cutting times when the number of discrete points was a constant value. The form error is not significantly improved when the number of discrete point is greater than 400. Taking the machining efficiency into account, the number of discrete points is 400 for a single micro lens. The cutting time of every discrete point is 0.5, 1, 1.5, 2, and 2.5 ms. The plot of form error of machined micro lens with different cutting times of every discrete point is shown in Figure 12. It can be seen that increasing the cutting time has a certain impact on improving form error of micro lens. When the cutting time of every discrete point is 0.5 ms, RMS is 0.746 μm. As the cutting time of every discrete point increases, RMS decreases slightly. Furthermore, when the cutting time of every discrete point is 1.5, 2, and 2.5 ms, RMS are 0.632, 0.651, and 0.626 μm, respectively.

This is because increasing the cutting time of every discrete point can further reduce acceleration and deceleration of the cutting axis when the cutting depth is the same value. In addition, fluctuations of follow error of the cutting axis also further decrease correspondingly. Thus, the surface quality of micro lens can be improved further. Similarly, too much cutting time of every discrete point do not decrease form error significantly.

### 4.4. Effect of Cutting Depth

**Figure 13:**
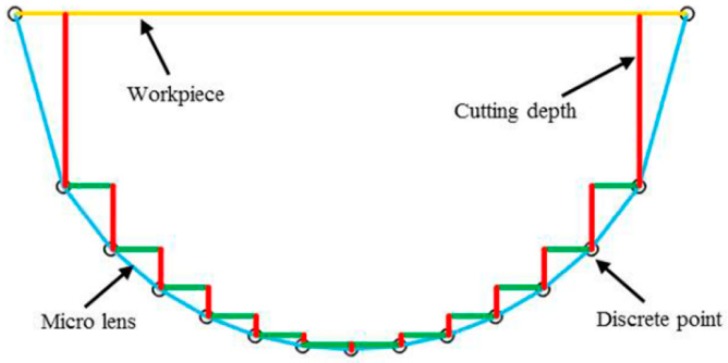
Schematic of the change trend of cutting depth for the equal-angle method.

The amplitude of the form error is greater at the cut-in part and cut-off part in comparison to other cutting areas along the cutting direction. It is an obvious feature in the 2D sectional profile of the form error of machined micro lens surface, as shown in Figure 8b and Figure 11b. This is because cutting depth of every discrete point is different when the equal-angle method is used during processing, as shown in Figure 13. The maximum value of cutting depth occurs at the first and last discrete point; the minimum value of cutting depth appears at the bottom of every micro lens. With the increasing cutting depth, the cutting force is also bigger. Certainly, the cutting force will determine the elastic recovery of materials and relieving amount, i.e., bigger cutting force generates greater elastic recovery and relieving amount after cutting [29]. The form accuracy of micro lens will degrade due to the existence of elastic recovery and relieving amount. Therefore, the form error at the cut-in part and cut-off part is larger under the influence of bigger elastic recovery and relieving amount.

**Figure 14:**
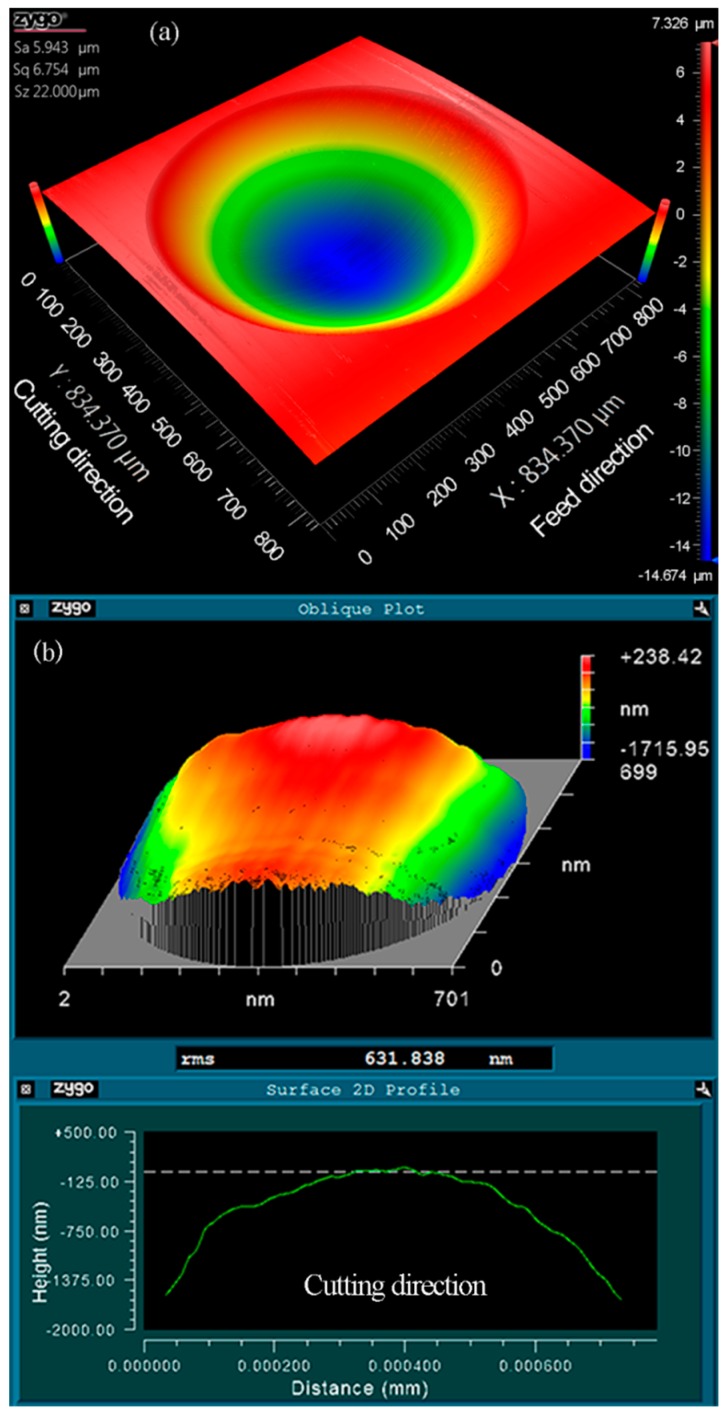
Zygo photographs of micro lens. (**a**) 3-D topography of machined micro lens; (**b**) The form error of machined micro lens surface from a designed sphere (*n* = 400, *t* = 1.5 ms).

Based on the discussion above, the optimal cutting parameters were adopted for generating typical MLAs (20.52-μm height and 700-μm aperture), which took into account form accuracy, as well as machining efficiency. The number of discrete points was 400, and the cutting time of every discrete point was 1.5 ms. The measurement result is shown in Figure 14 with 0.632 μm RMS and no chatter mark was observed. Compared with the previous research, both aperture and height of the machined micro lens vary from different researchers. So, RMS cannot be directly compared to evaluate the form error of micro lens. In terms of chatter mark, the acquired surface quality is similar to or even better than the conventional trajectory method based on FTS/STS diamond turning of MLAs [21,28]. Therefore, the experimental result proves the effectiveness of the novel forming method based on STS diamond turning. The extendable fabrication of similar microstructure surfaces can be also performed in this way.

## 5. Conclusions

A novel forming method based on STS diamond turning is presented to generate MLAs on the roller mold, which addresses the inherent drawback of relatively low production efficiency associated with traditional trajectory method. The MLAs (20.52-μm height and 700-μm aperture) is successfully machined without a chatter mark using the optimal cutting parameters. The proposed method can be extensively applied to other microstructures. According to the above experimental results, the conclusions can be presented as follows:

1. Taking into account the capacity of the control and motor system, the equal-angle method is recommended during processing. In this tool path, there is no frequent speed changes of the C-axis in the case of the heavy load during processing;
2. According to the kinematic analysis of the cutting axis, the chatter mark can be attributed to the overlarge instantaneous acceleration oscillations of the cutting axis of the machine tool during STS diamond turning of MLAs;
3. Increasing the number of discrete points of every micro lens will reduce the form error effectively. Furthermore, when the number of discrete points is greater than 400, MLAs have a fine surface quality without the chatter mark. And when the discrete point is 800, the form error of machined micro lens is reduced by about 31.88%, i.e., RMS is 0.686 μm;
4. Increasing the cutting time of every discrete point also has a certain impact on improving the surface quality of micro lens. When the cutting time of every discrete point is 2.5 ms, the form error of machined micro lens is reduced by about 37.84%, i.e., RMS is 0.626 μm. But too much cutting time of every discrete point does not decrease form error significantly.

## 6. Future Work

In the current study, although the forming method can be used for generating the MLAs with no chatter mark efficiently. As for the form accuracy of the machined micro lens, the effect of elastic recovery and relieving amount for the form accuracy cannot be neglected. If both factors can be studied and compensated, the form accuracy is likely to be improved further. In the future research, compensation strategy of form accuracy will become the focus based on the forming method. 

## Figures and Tables

**Table 1:** Specification of the machine tool.

The Machine Tool	Values
Positioning accuracy	C-axis: ±3 arc s (compensated)
	X-axis: 0.73 μm/200 mm (compensated)
	Z-axis: 0.95 μm/1100 mm (compensated)
Repetitive positioning accuracy	C-axis: ±2 arc s (compensated)
	X-axis: 0.63 μm/200 mm (compensated)
	Z-axis: 0.88 μm/1100 mm (compensated)

**Table 2:** Experimental conditions for generation of micro lens arrays (MLAs).

The Cutting Tool	Values
Tool material	Single-crystal diamond
Tool nose radius *r_t_*	2.995 mm
Tool rake angle *α_t_*	0°
Tool clearance angle *γ_t_*	8°
**The Cutting Parameters**	
Number of discrete points *n*	100, 200, 400, 600 and 800
Cutting time of every discrete point *t*	0.5, 1, 1.5, 2 and 2.5 ms
Workpiece material	Aluminum alloy (6061)
Lubricant	ISOPAR H
**The Size of Micro Lens**	
Height of the micro lens	20.52 μm
Aperture of micro lens	700 μm
The pitch of radial direction	1 mm
The pitch of axial direction	1 mm
